# Recess Physical Activity and Perceived School Environment among Elementary School Children

**DOI:** 10.3390/ijerph110707195

**Published:** 2014-07-15

**Authors:** Kaori Ishii, Ai Shibata, Mai Sato, Koichiro Oka

**Affiliations:** 1 Faculty of Sport Sciences, Waseda University, 2-579-15 Mikajima Tokorozawa, Saitama 359-1192, Japan; E-Mails: shibata@taiiku.tsukuba.ac.jp (A.S.); koka@waseda.jp (K.O.); 2 Faculty of Health and Sport Sciences, University of Tsukuba, 1-1-1 Tennodai, Tsukuba, Ibaraki 305-8574, Japan; 3 Graduate School of Sport Sciences, Waseda University, 2-579-15 Mikajima Tokorozawa, Saitama 359-1192, Japan; E-Mail: smai1002@yahoo.co.jp

**Keywords:** children, school, recess, physical activity, environment

## Abstract

Differences in recess physical activity (PA) according to perceived school environment among elementary school children were examined. Participants were 103 children from two schools in Japan. PA was measured using accelerometry for seven consecutive days. Time spent in sedentary or PA (light, moderate, or vigorous) during their morning recess (25 min) and lunch recess (15 min) was determined. The School Physical Activity Environment Scale (three factors: equipment, facility, and safety) was used to investigate perceived school environment. Environmental factor scores were assigned to low or high groups for each factor by median. An analysis of covariance, with grade as the covariate, was conducted separately by gender to examine differences in PA between two groups. During lunch recess, boys in the high-equipment group spent significantly more time in moderate PA (high: 1.5; low: 0.8 min) whereas girls in this group spent less time in light PA (9.3, 11.0). Boys in the high-facility group spent significantly less time in sedentary (2.3, 3.9) and more time in vigorous PA (2.4, 1.4) during lunch recess, and girls spent more time in moderate (2.1, 1.2) and vigorous PA (1.9, 1.3) during morning recess. Differences were observed in recess PA according to school environment perceptions. The present study may be useful for further intervention studies for the promotion of PA during recess.

## 1. Introduction

Physical activity during childhood is important for physical and mental health [1,2,3]. However, previous research has found that the level of physical activity in children has decreased over the last several decades in numerous industrial countries [4]. Therefore, the promotion of physical activity among children is an important public health challenge.

To promote physical activity among children, various interventions have been conducted at the home, community, and school levels [5]. In particular, the school has been identified as one of the key settings for promoting physical activity of children [6], because children spend the greater part of their days at school. In contrast to physical education classes or after-school hours, recess could be an ideal opportunity for promoting daily physical activity [7]. In Japan, recess is scheduled every day, and the same duration is provided for all children. Recently, recess has been shown to contribute up to 40% of daily physical activity recommendations [8]. In addition, a threshold of 40% of physical activity during recess has been proposed for children [9]. However, the proportion of time spent in moderate-to-vigorous-intensity physical activity during recess was recently found to be only 18% among Japanese children [10]. According to previous studies conducted in other countries, the proportion of time spent in moderate-to-vigorous-intensity physical activity during morning and lunch recess was reported to be 32.1% in boys and 23.7% in girls in France [11] and 32.9% in boys and 25.3% in girls in England [12]. Compared to these studies, the levels of physical activity observed during school recess are low in Japan. Thus, effective recess-time strategies to promote physical activity among children must be identified.

Recent evidence has indicated that the environment affects physical activity among children [13]. Previous studies in Europe and Australia found that recess physical activity was associated with aspects of the school physical environment, such as large play space [14], adequate equipment [14,15,16,17,18,19,20], and playground marking [19,21,22]. In addition, playground-related environmental interventions such as equipment provision, multicolor marking, and clear division by activity type have been reported to be effective in promoting physical activity during recess [23,24,25,26,27]. These studies have also found that gender differences play a role in the effectiveness of the interventions. This suggests that it may be important to focus on the association between perceived environment and recess physical activity separately by gender when considering effective strategies for promoting physical activity during recess in order to understand environmental factors that should be addressed differently for boys and girls.

Haug *et al.* [28] suggest that the physical environment factors that affect physical activity during recess differ by culture or lifestyle of countries, however most previous research has been conducted outside Japan. In a previous study, Lee *et al.* [29] pointed out that, even if the objectively measured actual environmental factors were the same, physical activity varied depending on the perception of these environmental factors by the people involved who carried out these measurements subjectively. However, school environments were evaluated by the researcher, principal, or teachers [19,21]. Their perceptions may not be always matched with those of children; therefore, the association between recess physical activity and perceived school environment in Japan must be confirmed. Investigating the association between children’s own evaluation of their perceived environments and recess physical activity would enable the identification of those environmental factors that should be the focus of efforts to promote physical activity during recess time, which offers daily opportunities for children to engage in physical activity. The present study examines the association of perceived school environment with the level of physical activity during recess among Japanese elementary school children.

## 2. Methods

### 2.1. Participants and Procedures

The present cross-sectional study was conducted in 2010. Two hundred and thirty children (127 boys and 103 girls) aged 6–12 years were recruited from two public elementary schools who agreed to the survey in Saitama prefecture, Japan. After permission to conduct the present study was received from the principals of each school through the Board of Education of the city, children and their parents/guardians were sent a letter explaining the ethical considerations of the study, the questionnaire, and an accelerometer. Return of the questionnaire meant that parents/guardians understood the letter and gave their informed consent (consent rate was 88.8%). Children received the questionnaire, accelerometer, and return envelope from their class teachers. They were instructed to answer the questionnaire at home and return it to the classroom teacher, using the envelope. For children from the first to third grades, the parent/guardian answered the questionnaire by discussing questions with the child. Children from the fourth to sixth grades responded to the questionnaire without parental/guardian assistance, according to previous studies [30]. The present study was approved by the human research ethics committee at Waseda University (application number 2010-234).

### 2.2. Measures

***Demographic data:*** Data on gender, age, height and weight which were stored in school were reported from classroom teacher. Body mass index (BMI; kg/m^2^) was calculated from the height and weight data.

***Physical activity:*** Physical activity was measured for seven consecutive days, using accelerometers (Lifecorder, SUZUKEN CO., LTD., Japan). This accelerometer is a waist-worn monitor that has been widely used by individuals from children to the elderly in Japan. Previous study among Japanese children reported that physical activity energy expenditure using the doubly labeled water method was significantly correlated with physical activity intensity using Lifecorder [31]. The accelerometer epoch length was 2 min. Children were instructed to wear the accelerometer throughout the day except during sleep and water-related activities (e.g., bathing, swimming). Physical activity during morning recess (between the second and third classes; approximately 25 min) and lunch recess (after lunch: 15 min) was evaluated. These recesses were scheduled at the same time every day and were of the common length of recess periods in Japan. During recess, children did not require access to the playground. On the basis of the nine exercise intensity levels from the Lifecorder data, physical activity was classified into sedentary time (0–0.5; <1.5 Mets), or light- (1–3; ≥1.5 to <3 Mets), moderate- (4–6; ≥3 to <6 Mets), or vigorous- (7–9; ≥6 Mets) intensity physical activity. The average time spent in each intensity level was determined. Children with data showing continuous zero counts for the complete recess period were excluded, and participants were included for analysis if they had complete data on a minimum of three days. The Lifecorder data were edited and aggregated using the Data Conversion Software (Knack Technical System, Inc.).

***School environment:*** The School Physical Activity Environment Scale was used [30]. The scale is composed of 10 items from three factors: equipment, facility, and safety. It has been confirmed to have acceptable construct validity (factor loading: equipment = 0.617–0.901, facility = 0.512–0.919, safety = 0.568–0.838), internal consistency (equipment, α = 0.87; facility, α = 0.84; safety, α = 0.86) and inter-rater reliability (equipment, *r* = 0.524; facility, *r* = 0.677; safety, *r* = 0.515). The equipment factor consists of three items: “the equipment at my school is easy to use for engaging physical activity and sports (e.g., horizontal bar, and ball), “my school has enough equipment that I can use actively,” and “my school has enough equipment that I can use actively during recess period.” The facility factor includes four items: “my school’s grounds are easy to use,” “my school’s gym is easy to use,” “my school’s grounds are large enough to allow me to be active,” and “my school’s gym is large enough so that I can spend time their being active.” The safety factor has three items: “the grounds and gym at my school are safe to use,” “the school facilities are safe to use for engaging physical activity,” and “my school’s gym and grounds are well-maintained.” Participants chose responses on a 4-point scale (1: “strongly disagree” to 4: “strongly agree”). Responses were scored for each factor, and dichotomized into high or low groups on the basis of the median for each. Generally in Japan, there are facilities such as slides, exercising bars, climbing frames, seat swings, and sandboxes on the school grounds, and equipment such as different kinds of balls, unicycles, and jump ropes are provided during recess. Moreover, all children in the same school were provided with the same environment, same time, and same area for play. Similar environments were provided for the schools in the present study.

### 2.3. Statistical Analyses

An independent t-test was performed to examine gender differences in the demographic data and physical activity. Additionally, a one-way analysis of covariance adjusted grade as the covariate, was conducted separately for boys and girls to determine differences in time spent in sedentary time, or in light, moderate, or vigorous physical activity between the high and low environment groups. All statistical analyses were performed using PAWS Statistics 18 and results were considered significant at *p* < 0.05.

## 3. Results

### 3.1. The Characteristics of the Participants

Of the 230 children who were recruited, 103 children (64 boys and 38 girls) responded to the questionnaire (response rate: 44.3%). Of these, 68 children (39 boys and 29 girls) during morning recess and 69 children (40 boys and 29 girls) during lunch recess had valid physical activity data. These children formed the final sample. The mean ± SD values of age, height, weight, and BMI were 9.2 ± 1.6 years, 133.1 ± 11.8 cm, 30.2 ± 7.7 kg, and 16.8 ± 2.3 kg/m^2^, respectively. There were no significant gender differences between these variables. During morning recess and lunch recess, total minutes spent in physical activity were as Table 1. Gender differences were found for light PA (*p* < 0.10) and vigorous PA (*p* < 0.01) in morning recess and vigorous PA (*p* < 0.10) in lunch recess. There are no significant differences in all physical activity between children at each school. The median score (25th through 75th percentile) of each factor is 8.9 (8.1–9.8) for equipment, 11.8 (10.8–12.8) for facility, and 9.3 (8.4–10.1) for safety.

**Table 1:** Descriptive characteristics by gender (numbers and percentages).

		Boys			Girls		
		Mean	±	SD	Mean	±	SD
Age		9.1	±	1.7	9.3	±	1.5
Height		131.4	±	10.5	135.4	±	13.2
Weight		29.7	±	7.6	31.0	±	8.0
BMI		16.9	±	2.4	16.6	±	2.1
PA in morning recess		*n* = 39			*n* = 29		
	Sedentary	7.5	±	4.5	9.0	±	4.4
	Light PA	12.1	±	2.8	13.7	±	3.9
	Moderate PA	1.7	±	1.1	1.7	±	1.1
	Vigrous PA	4.7	±	3.4	1.6	±	1.0
PA in lunch recess		*n* = 40			*n* = 29		
	Sedentary	2.8	±	2.3	3.2	±	2.2
	Light PA	9.8	±	2.0	9.8	±	2.3
	Moderate PA	1.4	±	0.8	1.5	±	0.8
	Vigrous PA	2.1	±	1.7	1.4	±	1.3

PA: physical activity, SD: standard deviation, numbers in Table 1 are rounded.

### 3.2. Differences in Recess Physical Activity according to Perceived School Environment

Table 2 and Table 3 present gender differences in time spent in sedentary, or light, moderate, or vigorous physical activity between high and low environment groups. Among boys, the high-equipment group engaged in significantly more moderate physical activity than low-equipment group during lunch recess [F(1, 37) = 4.30, *p* = 0.05]. The high facility group had significantly less sedentary [F(1, 37) = 4.54, *p* = 0.04] and high vigorous physical activity [F(1, 37) = 4.63, *p* = 0.04] during lunch recess (Table 2). Among girls, the high-equipment group engaged in significantly less light physical activity than low-equipment group during lunch recess [F(1, 26) = 4.30, *p* = 0.05]. Furthermore, the high-facility group engaged in more moderate physical activity [F(1, 26) = 4.06, *p* = 0.05] and vigorous physical activity [F(1, 26) = 5.44, *p* = 0.03] during morning recess than low-facility group (Table 3). The remaining variables did not significantly differ between the high- and low-facility groups.

**Table 2:** Differences in recess physical activity according to perception of school environment (boys).

	Equipment				Facility				Safety			
	High	Low	F	*p*	High	Low	F	*p*	High	Low	F	*p*
	Mean, SD	Mean, SD			Mean, SD	Mean, SD			Mean, SD	Mean, SD		
Morning Recess (*n* = 39)												
Sedentary	7.5, 4.4	7.7, 5.4	0.00	0.98	7.6, 4.2	7.5, 5.6	0.01	0.92	7.3, 4.4	8.3, 5.4	0.15	0.70
Light PA	12.1, 2.6	12.0, 3.7	0.00	0.97	12.1, 2.6	12.1, 3.2	0.00	0.96	12.0, 2.7	12.3, 3.1	0.27	0.61
Moderate PA	1.6, 1.1	2.2, 1.4	1.99	0.17	1.7, 1.1	1.8, 1.4	0.15	0.71	1.8, 1.2	1.4, 1.0	0.27	0.61
Vigorous PA	4.8, 3.5	4.2, 3.4	0.20	0.66	4.7, 3.0	4.7, 4.5	0.00	0.98	4.9, 3.5	4.0, 3.0	0.58	0.45
Lunch Recess(*n* = 40)												
Sedentary	2.5, 2.0	3.9, 3.1	2.61	0.11	2.3, 1.7	3.9, 3.1	4.54	0.04	2.6, 2.1	3.3, 3.0	1.30	0.26
Light PA	9.8, 2.0	9.8, 2.4	0.00	0.99	9.8, 2.0	9.7, 2.3	0.04	0.84	9.7, 2.1	9.9, 1.9	0.08	0.78
Moderate PA	1.5, 0.8	0.8, 0.6	4.30	0.05	1.4, 0.8	1.1, 0.8	1.32	0.26	1.4, 0.8	1.2, 0.8	0.02	0.88
Vigorous PA	2.2, 1.8	1.5, 1.3	1.66	0.21	2.4, 1.8	1.4, 1.3	4.63	0.04	2.3, 1.8	1.5, 1.5	3.92	0.06

PA: physical activity, SD: standard deviation, one-way analysis of covariance were performed, adjusted for grade

**Table 3:** Differences in recess physical activity according to perception of school environment (girls).

	Equipment				Facility				Safety			
	High	Low	F	*p*	High	Low	F	*p*	High	Low	F	*p*
	Mean, SD	Mean, SD			Mean, SD	Mean, SD			Mean, SD	Mean, SD		
Morning Recess(*n* = 29)												
Sedentary	9.0, 4.0	9.1, 5.5	0.00	0.95	8.1, 4.2	10.1, 4.6	0.81	0.38	8.7, 4.6	10.2, 3.7	0.23	0.63
Light PA	13.6, 3.4	14.0, 5.0	0.07	0.79	13.9, 3.8	13.4, 4.1	0.01	0.94	14, 4.0	12.6, 3.6	0.22	0.64
Moderate PA	1.7, 1.1	1.7, 1.0	0.03	0.86	2.1, 1.2	1.2, 0.6	4.06	0.05	1.8, 1.1	1.6, 0.8	0.01	0.94
Vigorous PA	1.7, 1.0	1.3, 1.0	1.27	0.27	1.9, 1.0	1.3, 1.0	5.44	0.03	1.6, 1.1	1.7, 1.0	0.04	0.84
Lunch Recess (*n* = 29)												
Sedentary	3.6, 2.4	2.2, 1.4	3.57	0.07	3.6, 2.3	2.7, 2.2	0.02	0.90	3.4, 2.2	2.5, 2.6	0.05	0.83
Light PA	9.3, 2.2	11.0, 2.2	4.30	0.05	9.2, 1.9	10.7, 2.6	1.57	0.22	9.7, 2.3	10.5, 2.4	0.09	0.77
Moderate PA	1.6, 0.8	1.4, 0.8	0.34	0.56	1.7, 0.9	1.3, 0.6	0.93	0.34	1.5, 0.9	1.4, 0.6	0.00	0.95
Vigorous PA	1.5, 1.4	1.4, 1.1	0.04	0.84	1.5, 1.3	1.3, 1.2	2.40	0.13	1.4, 1.3	1.7, 1.3	0.05	0.83

PA: physical activity, SD: standard deviation, one-way analysis of covariance were performed, adjusted for grade

## 4. Discussion

The present study investigated the effect of perceived school environment on physical activity during recess among elementary school children. Boys who perceived school equipment for physical activity to be good engaged in more moderate physical activity during lunch recess. Moreover, boys who perceived that the school had good facilities for physical activity engaged in higher vigorous physical activity and less sedentary time during lunch recess. Similarly, girls who perceived the school facilities for physical activity to be good spent longer time in moderate and vigorous physical activity during their morning recess. Girls who perceived school equipment for physical activity to be good engaged in light physical activity for a shorter duration. The present results revealed which environmental factors should be addressed in order to promote physical activity in recess time, which offers daily opportunities for children to engage in physical activity. Moreover, physical activity during recess differs by gender according to perceived school environment. In order to promote physical activity during recess more effectively, working to increase perception of equipment and facilities may be important for boys during lunch recess, and to increase perception of facilities may be important for girls during morning recess.

Previous research has indicated that play equipment available at school is related to children’s physical activity during recess times [13,32]. Previous studies have found that a larger proportion of students participated in moderate physical activity during recess when fixed play equipment was provided [19,21,28,33], and that the number of permanent play devices in the playground was positively associated with the amount of physical activity [34]. The present study demonstrated that boys who perceived school equipment to be good engaged in more moderate physical activity during lunch recess, which supports the findings of previous studies. However, girls who perceived school equipment to be good spent less time in light physical activity during lunch recess. While previous research has demonstrated gender differences regarding certain characteristics of recess physical activity [18,35], there may also be gender differences in regard to equipment preferences. Moreover, girls who perceived school equipment to be good spent a long time being sedentary during lunch recess, though this was not significant. The reason for this is unclear. However, in order to reduce sedentary time, it may be effective to engage in efforts to improve perceptions regarding school equipment, and then assess whether sedentary time increases or decreases, as a result. If perceptions regarding school equipment are found to increase sedentary time, then it may be beneficial to focus on school environment factors other than those perceptions. However, the physical activity differences observed between the high and low perceived equipment groups is small. In Japan, recess time is provided daily to all children as an opportunity for them to engage in physical activity. This means that, although the time spent in physical activity between the high and low perceived equipment groups is short, it may add up to a major time difference. Further research is needed to determine gender equipment preferences and how these influence physical activity during recess.

Regardless of gender, children who perceived school facilities for physical activity to be good were more physically active during recess, especially vigorous physical activity. This finding partly supports previous research. In the present study, facility perceptions were assessed from questions regarding the “size” and “usability” of school grounds or gymnasium. Ridgers *et al.* [14] showed that as play space per child increased, time spent being sedentary decreased and vigorous physical activity increased. This positive association was also found in another study [36]. Willenberg *et al.* [19] observed that bitumen areas with markings/goals and play lines were associated with a higher proportion of active children than those without. Previous British and Australian studies have also found that multicolor markings and division by activity type were effective in increasing physical activity during recess among elementary children [16,24,27]. Such court markings or field lines may be one important aspect that influences the “usability” of school grounds among children. Moreover, other factors such as school policy or ground surface conditions can also affect “usability.” However, although the existence of court markings and field lines may enable children themselves to choose physical activity, school policy is not a matter that lies within their jurisdiction. If it is deemed necessary to improve the perception of usability in order to promote physical activity during children’s recess time, therefore, it may be important to focus on matters that lie within the scope of children’s own choices. The present results show that environment interventions to enhance perception of “usability” and “size” would be effective in promoting physical activity during recess. For example, effective utilization of spaces by line marking or area division can potentially enhance the perceptions of “usability” and “size” even without changing the physical size of school grounds or gymnasium.

There were no differences in physical activity according to the perception of safety between genders. This finding implies that for both boys and girls, perceptions of school safety and maintenance may have a minimal impact on physical activity during recess. Previous studies have conceptualized “safety” in terms of play space size, teacher supervision, and surface type (grass or bitumen, soft or hard) [19,36]. As a result, some of these variables were identified as factors that promote physical activity. However, most environmental variables, including safety variables, have been assessed by researchers or teachers, and no studies have explored how children’s perceptions of the school environment are associated with their physical activity during recess. Therefore, it might not be easy to compare the present data with previous findings. The inclusion of multiple factors of “safety” may partly explain the weak association between safety factors and physical activity during recess. For instance, Parrish *et al.* [37] asked children and teachers about how school environment affected physical activity during recess, and both believed that children who are lightweight or have fundamental movement skills lower than those of their peers do not want to join games, owing to bullying. Therefore, “safety” may involve not only the currently studied variables but also social environment factors (e.g., teacher supervision and school policy), interpersonal factors (e.g., self-efficacy, activity preference, fundamental activity skills, and clothes), and weather. Additional research is needed to explore the association between various understandings of “safety” and physical activity.

In the present study, children who perceived the school equipment and facilities to be good were physically active. The relationship between school facilities, equipment, and physical activity may be bi-directional; children who seek out activity opportunities are more aware of the available equipment and other facilities. This finding suggests that an approach focused on improving equipment and facilities would be effective in increasing physical activity among children. However, environment factors associated with physical activity did not have the same impact for boys and girls, or during morning recess and lunch recess times. This observed difference could be explained by the theory that boys and girls use space and time differently; other research has found that boys spend more time engaging in sport and competition with a larger group, using extensive space, whereas girls consider recess an opportunity to socialize and engage in sedentary play in smaller groups [38]. Gender differences associated with school environment factors and physical activity during recess may have resulted from gender preferences in the type of activity contents. There were differences in the amount of physical activity among boys during lunch recess and girls during morning and lunch recess, depending on the school environment. This gender difference could be due to the different duration of lunch and morning recess, at 15 min and 25 min, respectively. This means that the children engage in different types of activity. Since different types of activity involve the use of different equipment and facilities, the observed gender difference may have stemmed from variations in the perception of these school environments. The present study is the first study to investigate differences in recess physical activity according to perceived school environment among Japanese elementary school children, and the strength of present study is that it has considered children’s subjective perceptions of their school environment.

Some limitations of the current study should be considered. First, the analysis was cross-sectional, thereby precluding causal inference. Second, the present study used an accelerometer with an epoch of 2 min. This accelerometer was only capable of measurements for 2-min epochs. A short epoch is strongly recommended for the measurement of children’s physical activity as such activity is often intermittent [39]. Therefore, the amount of sedentary time and vigorous physical activity might be underestimated, while light and moderate physical activity might be overestimated. Finally, the sample was small as children from only two schools participated, and therefore, the present study might have a limited generalizability. Despite these limitations, the present study is meaningful as it is the first to indicate differences in physical activity during recess as being associated with perceived school environment among Japanese children. The present data can be used to inform interventions for promoting physical activity in Japanese children during recess.

## 5. Conclusions

Differences were observed in recess physical activity according to perceptions about their school environment, but this tendency differed between boys and girls. This finding indicates the necessity of employing different approaches to promote physical activity by gender. The present data may be useful for further intervention studies and the promotion of physical activity during recess.
